# Self-help guided by trained lay providers for generalized anxiety disorder in older adults: study protocol for a randomized controlled trial

**DOI:** 10.1186/s12877-021-02221-x

**Published:** 2021-05-22

**Authors:** Philippe Landreville, Patrick Gosselin, Sébastien Grenier, Pierre-Hugues Carmichael

**Affiliations:** 1 School of Psychology, Université Laval, 2325 rue des Bibliothèques, Quebec City, Quebec G1V 0A6 Canada; 2 Centre d’Excellence sur le Vieillissement de Québec, Quebec City, Canada; 3 VITAM – Centre de Recherche en Santé Durable, Quebec City, Canada; 4 Centre de Recherche du Centre Hospitalier Universitaire de Québec-Université Laval, Quebec City, Canada; 5 Department of Psychology, Université de Sherbrooke, Sherbrooke, Canada; 6 Institut Universitaire de Première Ligne en Santé et Services Sociaux (IUPLSSS), Sherbrooke, Canada; 7 Department of Psychology, Université de Montréal, Montreal, Quebec Canada; 8 Centre de Recherche de l’Institut Universitaire de Gériatrie de Montréal (CRIUGM), Montreal, Canada

**Keywords:** Generalized anxiety disorder, Older adults, Guided self-help, Cognitive-behavioral therapy, Lay providers, Randomized controlled trial

## Abstract

**Background:**

Only a small proportion of older patients with generalized anxiety disorder (GAD) seek professional help. Difficulties in accessing treatment may contribute to this problem. Guided self-help based on the principles of cognitive-behavioral therapy (GSH-CBT) is one way of promoting access to psychological treatment. Moreover, because the therapist’s role in GSH-CBT is limited to supporting the patient, this role could be assumed by trained and supervised lay providers (LPs) instead of licensed providers. The main goal of this study is to evaluate the efficacy of GSH-CBT guided by LPs for primary threshold or subthreshold GAD in older adults.

**Methods:**

We will conduct a multisite randomized controlled trial comparing an experimental group receiving GSH-CBT guided by LPs (*n* = 45) to a wait-list control group (*n* = 45). Treatment will last 15 weeks and will be based on a participant’s manual. Weekly telephone sessions with LPs (30 min maximum) will be limited to providing support. Data will be obtained through clinician evaluations and self-assessment questionnaires. Primary outcomes will be the tendency to worry and severity of GAD symptoms. Secondary outcomes will be anxiety symptoms, sleep difficulties, functional deficit, diagnosis of GAD, and cognitive difficulties. For the experimental group, measurements will take place at pre- and post-treatment and at 6 and 12 months post-treatment. For the control group, three evaluations are planned: two pre-treatment evaluations (before and after the waiting period) and after receiving treatment (post-treatment). The efficacy of GSH-CBT will be established by comparing the change in the two groups on the primary outcomes.

**Discussion:**

This project will provide evidence on the efficacy of a novel approach to treat GAD in older adults. If effective, it could be implemented on a larger scale and provide many older adults with much needed mental health treatment through an expanded workforce.

**Trial registration:**

The trial was registered at ClinicalTrials.gov, number NCT03768544, on December 7, 2018.

## Background

Generalized anxiety disorder (GAD) is mainly characterized by excessive anxiety and worries that are difficult to control, occurring most days for at least 6 months, and are related to a variety of symptoms and activities. Anxiety and worries are associated with three or more of the following symptoms: agitation or feeling overexcited or fed up, tiredness, difficulty concentrating or memory gaps, irritability, muscle tension and sleep disorders [1]. GAD is one of the most common anxiety disorders in older adults. A 4.6% six-month prevalence rate of GAD has been found in community-dwelling older adults, with the first episode beginning after age 50 in one-quarter of cases [2]. Some researchers have found prevalence rates as high as 7.3% [3]. Subthreshold GAD, typically identified in studies when almost all but a few symptoms are present to meet the diagnostic threshold, is even more prevalent than threshold GAD [4]. The negative impact of GAD in late life, even at a subthreshold level, is significant and includes more disability, poorer quality of life and greater use of health care services [5–7]. Anxiety in older adults often presents with comorbid depression [8] and is associated with insomnia [9], poorer cognitive functioning [10], cardiovascular disease [11], greater suicide risk [12], and higher costs for health care [13].

Despite its deleterious consequences and the availability of effective pharmacological and psychological treatments [14], only a small proportion of older patients with GAD seek and use professional help [15, 16]. Various factors, including limited access to mental health care services, may contribute to the undertreatment of older persons [17]. Older adults are particularly likely to have transportation problems [18], as are those receiving home care services [19] and those living in rural areas who also face a limited supply of services [17]. Travel problems are accentuated by harsh climate and in countries, such as Canada, where large distances often have to be covered to get to services. Problems of access to care services are especially problematic for psychotherapy which typically requires weekly meetings with a psychotherapist. Indeed, travel distance is one of the variables significantly related to the use of psychotherapy by older adults [20].

Guided self-help (GSH) is one way of promoting access to psychological treatment. GSH has been defined as a psychological treatment where the patient takes home a standardized psychological treatment and works through it more or less independently [21]. Whereas psychotherapy is typically based on regular personal interaction with the therapist, in GSH (a) the patient uses step-by-step instructions on how to apply a psychological treatment procedure to himself, (b) the role of the therapist is primarily of supportive or facilitative nature, and (c) the amount of contact between the patient and therapist is minimized [21].

There is evidence, albeit limited, that GSH based on the principles of cognitive-behavior therapy (GSH-CBT) is effective for treating GAD in older adults. A randomized controlled trial by Dear et al. [22] evaluated an 8-week online guided program for older adults with anxiety symptoms. Participants were allocated to either the treatment group (*n* = 35) or to a waitlist control group (*n* = 37). Treatment material was presented in a didactic form combined with case-enhanced learning examples. Therapists attempted to contact participants each week via telephone or a secure internal email-type system and tried to limit contact to 5 to 10 min. Lower scores on measures of symptoms of GAD, assessed with the GAD-7 [23], and depression, assessed with the Patient Health Questionnaire-9 Item (PHQ-9 [24]), were found in the treatment group compared to the waitlist control group at post-treatment and were maintained at 3-month and 12-month follow-ups. Among participants in the treatment group, 16 initially had a diagnosis of GAD compared to only two at 3-month follow-up. The latter finding should be interpreted with caution due to the small number of participants with GAD. Moreover, this diagnosis was not a criterion for participation in the study, the authors did not report GAD data for participants in the control group, and diagnostic assessments were conducted by the therapists. Jones et al. [25] conducted a randomized controlled trial of a 10-week guided internet-delivered treatment for older adults with GAD (63% of the sample) or subclinical GAD. Participants were assigned to the treatment group (*n* = 24) or to a waiting list condition (*n* = 22). Those in the treatment group were provided psycho-educational material and CBT strategies for coping with GAD, including homework exercises to apply the skills. Therapist contact consisted of responding by e-mail to participants once weekly (each e-mail took approximately 15–30 min to compose) and served to provide support, guidance, advice, and encouragement. Faster improvements on the GAD-7 and PHQ-9 were observed for participants in the treatment group compared to those in the waiting list condition. A further reduction of scores on the GAD-7 but not on the PHQ-9 was observed at one-month follow-up. As a partial replication of the treatment effects observed in the randomized controlled trial, participants in the control group who eventually received treatment showed lower scores on the GAD-7 and PHQ-9 at post-treatment compared to post-waitlist but not from post-treatment to one-month follow-up.

Registered and experienced clinical psychologists served as therapists in the study by Dear et al. [22]. In the study by Jones et al. [25], the therapist was a graduate student under supervision who held a MA degree in clinical psychology and was eligible for registration in the province of Saskatchewan, Canada. Compared to psychotherapy, GSH requires less involvement from the therapist whose role is essentially to support the patient. This represents an advantage given that health care systems are struggling to adequately respond to the significant mental health needs associated with an aging population [26]. One of the proposed solutions to this problem is to increase the workforce capable of providing mental health care. In the United States, for example, the Institute of Medicine [26] and others [27, 28] have recommended the development of new models of care within existing services in order to expand their capacity to meet the mental health needs of the elderly. These include procedures for training and supervising an expanded workforce [26]. Thus, it is suggested that persons who are not licensed mental health professionals could be able to provide care under certain conditions with regards to training and supervision. Various terms, such as *paraprofessionals* and *non-licensed providers*, are used in the literature to refer to these persons. They are generally providers with no post-graduate training in a specialized mental health program [29]. In this article, we refer to them as lay providers (LPs).

Several studies have explored the effects of psychotherapy applied by LPs compared to professionals. Reviews of these studies show that LPs obtain positive results when they apply psychotherapy for anxiety and depressive symptoms and that these results are comparable to those obtained by professionals [29, 30]. Stanley et al. [31] conducted a randomized trial of CBT for GAD in older adults comparing bachelor-level LPs with Ph.D.-level expert providers as therapists. Patients who received CBT from either LPs or expert providers showed a greater improvement in the severity of GAD, anxiety, depression, and quality of life than patients receiving standard care. Importantly, improvements on these indicators of mental health did not differ between patients receiving CBT administered by LPs or expert providers and were maintained in both groups at 6- and 12-month follow-ups after the end of treatment [32]. Although the costs of training and supervising LPs are not negligible, the cost of treatment per patient has been shown to be much lower when it is administered by a LP rather than a professional such as a psychologist [27].

It is possible that GSH-CBT guided by LPs is effective for GAD in older adults but evidence is lacking. Our team conducted a single-case multiple-baseline study of GSH-CBT with three older adults who met criteria for GAD [33]. The intervention was based on the intolerance to uncertainty model of GAD developed by Dugas et al. [34] and the treatment protocol associated with this model [35]. Our choice of this protocol was based on its efficacy [36], particularly with older adults [37]. GSH-CBT targeted vulnerabilities of GAD, including intolerance to uncertainty, negative orientation to problems, cognitive avoidance, and the perception of the usefulness of worrying. The treatment also included two components, relaxation and behavioral activation, which are not part of Dugas et al’s protocol but can reduce anxiety and depression in older adults [38, 40]. Addressing depressive symptoms seems important considering that anxiety in older adults often presents with comorbid depression [8]. Participants used a manual presenting weekly readings and at-home practice exercises. They also received weekly supportive phone calls (maximum of 30 min) from a therapist who was a graduate student in clinical psychology with 2 years of supervised experience in CBT for anxiety disorders. Each phone session included a summary of the activities completed since the last session, answering participant’s questions and planning the next week’s activities. Results showed that following treatment, participants no longer met criteria for GAD and showed improvement on worries and GAD severity, on psychological variables targeted by the treatment (e.g., intolerance of uncertainty) and secondary variables associated with GAD (e.g., depression). These results were maintained up to 12 months after the end of treatment. Participants had favorable opinions toward the treatment. The therapist in this study, although not a licensed psychologist, cannot be considered a LP because of post-graduate training. Nonetheless, this preliminary work shows that GSH-CBT guided by a non-licensed provider is effective. Assuming that GSH-CBT guided by LPs is effective, it would be useful to determine for which patients such a treatment would be most effective and how it is perceived by both patients and LPs.

The main goal of this study is to evaluate the efficacy of GSH-CBT guided by LPs for generalized anxiety (i.e., threshold or subthreshold GAD) in older adults. Persons with subthreshold GAD will be included because of its high prevalence [4] and to reinforce the applicability of the findings to a broader and more representative clinical population. The project is a multisite randomized controlled trial comparing an experimental group receiving GSH-CBT guided by LPs to a wait-list control group. The project serves to verify that GSH-CBT is useful and effective and is a key preliminary step to future studies on this treatment, including a larger and more complex randomized controlled trial involving other treatment conditions. Specifically, this project aims to:

- Evaluate the effects of treatment on:
  i. GAD symptoms
  ii. The psychological mechanisms targeted by the treatment (intolerance to uncertainty, negative problem orientation, cognitive avoidance and perception of the usefulness of worrying)
  iii. GAD diagnosis and other variables associated with GAD (general symptoms of anxiety, depression, sleep difficulties, disability, and cognitive difficulties)
- Determine if treatment effects are maintained at 6 and 12 months post-treatment

The hypothesis is that the experimental group will show a significantly greater improvement than the wait-list group on GAD symptoms, as well as psychological mechanisms targeted by treatment and other variables associated with GAD.

Secondary goals are to identify variables (e.g., participants’ characteristics, motivation for entering treatment, credibility of treatment, expectancies about improvement, and working alliance between participants and LPs) associated with improvement during treatment and to document perception of treatment by participants and LPs.

## Methods

### Trial design

**Fig. 1:**
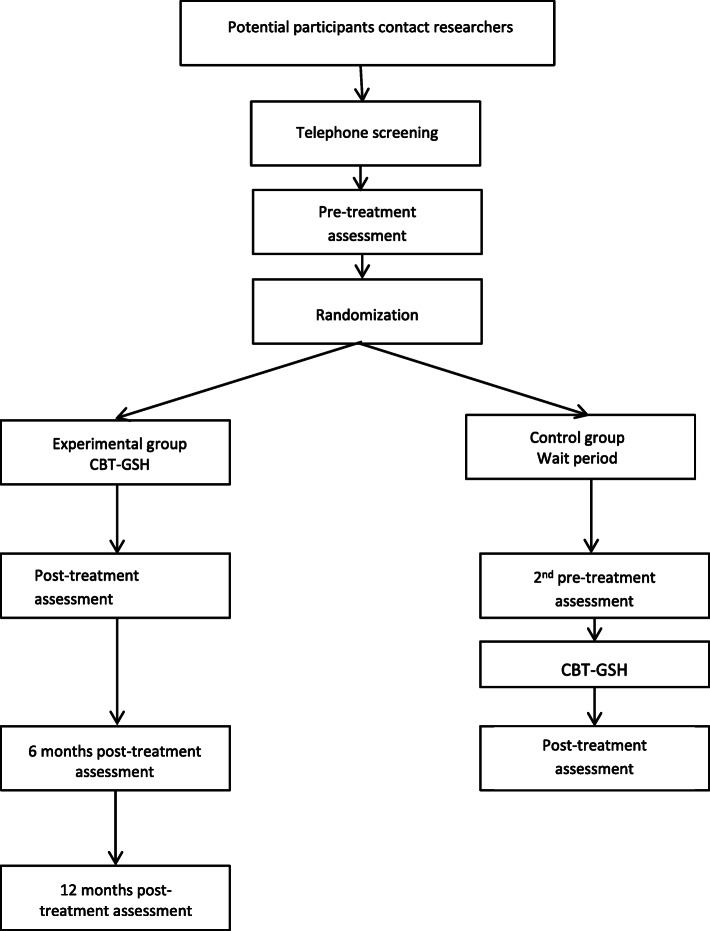
Schematic Representation of the Randomized Controlled Trial

This is a multisite randomized controlled trial comparing an experimental group receiving GSH-CBT guided by LPs to a wait-list control group. The control group will receive GSH-CBT after the waiting period. Because this is a wait-list condition, we will provide no active treatment to participants in this group during the waiting period. This will also maximize their response to GSH-CBT and allow us to include their data in analyses to identify characteristics associated with improvement during treatment. Data will be collected at pre-treatment, post-treatment and 6 and 12 months post-treatment. A multisite study will allow the recruitment of the required sample and increase the generalizability of the study. Université Laval will act as the study sponsor. The study will take place at three sites (Université Laval, Université de Sherbrooke, and Centre de recherche de l’Institut universitaire de gériatrie de Montréal) and patients at each site will be recruited into both intervention groups. A schematic representation of the randomized controlled trial is shown in Fig. 1.

### Participants

A power analysis was performed based on the Penn State Worry Questionnaire (PSWQ [39]), which is traditionally used as the primary measure of change in studies of CBT for GAD with older adults. Assuming an effect size of .65 (difference in standard deviations between the scores of the treatment group and the wait-list group with telephone support, based on the work of other researchers [41] and a conservative estimate), an alpha threshold of .05 and power of .80, 90 participants are required (45 in each group). In anticipation of an estimated 30% drop-out rate based on previous work [41], a total of 130 people will be admitted to the study (accepted for treatment) in order to obtain the required number of 90. The maximum proportion of participants with a diagnosis of subthreshold GAD will be set at 40% of the sample. This percentage is similar to that in the study by Jones et al. [25] and reflects the priority given to GAD.

In order to be included, interested individuals will have to (a) be 60 years of age or older, (b) meet Diagnostic and Statistical Manual of Mental Disorder fifth edition (DSM-5 [1]) criteria for primary threshold or subthreshold GAD, (c) be able to read and speak French and to use the telephone without difficulty, and (d) if an anxiety medication is used, commit to maintaining the type of medication or dose during 8 weeks prior to treatment and during the protocol. Criteria for subthreshold GAD are based partly on the work of Miloyan et al. [4] and are as follows: 1) anxiety and excessive worries (expectation with apprehension) concerning several situations or activities; 2) anxiety and worries occurring most of the time; 3) anxiety and worries for a period of at least 6 months; 4) presence of one or two of the following: a) difficulty controlling worries; b) at least 3 of the following symptoms: restlessness or feeling keyed up or on edge, being easily fatigued, difficulty concentrating or mind going blank, irritability, muscle tension, sleep disturbance; c) significant impairment and distress related to worries and anxiety; 5) the subject of worries and anxiety is not better explained by another mental disorder. Participants with another disorder will be eligible if the interference associated with their comorbid disorder is not a contraindication to an initial therapeutic approach targeting generalized anxiety or if their comorbid disorder does not require an immediate intervention. The following exclusion criteria will be used during recruitment since they are associated with variations in the level of anxiety or to difficulties in adherence to treatment: (1) a disabling physical disorder that is not adequately controlled (e.g., acute heart disease, recent stroke), (2) the presence of a substance use disorder, (3) presenting a bipolar disorder or symptoms of a psychotic disorder, (4) having significant cognitive impairment (score lower than 22 on the Telephone version of the Mini-Mental State Examination (T-MMSE [42]), (5) currently receiving or having received psychotherapy for worries or anxiety over the last 6 months. Withdrawal of a participant admitted to the study will be made at the participant’s request or if an unforeseen condition prevents him or her from continuing to participate in the study or benefiting from the treatment. In the case of a withdrawal, the participant will be asked to answer the measurement instruments one last time. For all participants, a form will be completed by the site’s professional research assistant indicating whether the protocol was followed and, if not, why not.

Recruitment will be done with a continuous, targeted and diversified campaign (e.g., posters in institutions attended by older adults, articles in newsletters, magazines, and regional newspapers, older person’ awareness conferences, media interviews, on the Internet). The three study sites are located in three administrative regions of the province of Quebec, Canada, with a total population of nearly 800,000 people aged 60 and over (Capitale-nationale: 221,766; Montréal: 457,370; Estrie: 102,072) [43]. This population is sufficient for recruiting the sample. Individuals living in other administrative regions will be also eligible.

### Measures

#### Initial telephone screening

A structured interview protocol will be used by the site’s professional research assistant to conduct a partial verification of participation criteria. The presence of at least subthreshold GAD at this point will be based on the presence of various worry themes and at least moderate scores on items of the Worry and Anxiety Questionnaire (WAQ [44]) assessing anxiety and worries, somatic symptoms, and their interference during the past 6 months. The WAQ is an 11-item self-report screening questionnaire that was developed for the rapid assessment of GAD. Items measure symptoms on a 9-point Likert scale with three qualifying statements (for the lowest, middle and highest ratings on the scale).

#### Telephone clinical psychological assessment

Eligible participants will be evaluated by independent research assistants (i.e., not LPs or researchers) experienced in clinical assessment. After initial training, they will be supervised by independent clinicians (i.e., not the researchers). Following the collection of socio-demographic data (age, sex, education, marital status, main occupation, residence, income, health status and place of birth), use of medication for anxiety will be assessed with questions about type and dose of drugs used in the past 3 months. Drugs classified as anxiolytics (e.g. benzodiazepines) or antidepressants (e.g. SSRIs) will be considered. Cognitive difficulties will be assessed using the T-MMSE. A diagnostic evaluation will be conducted using the Anxiety and Related Disorders Interview Schedule for DSM-5 (ADIS-5 [45]). The ADIS-5 is a structured interview used to confirm the presence or absence of anxiety disorders and associated psychiatric diagnoses. Various measures will be put in place to ensure that the diagnostic assessment is standardized and accurate: all assessors will receive the same training in the use of ADIS-5, supervision will be mandatory for the first two assessments and every five assessments thereafter, ad hoc supervision will be available and the supervisors will be the same for all sites. Each interview will be recorded on a digital audio device to allow for the calculation of interrater agreement.

#### Primary outcome measures

These measures will evaluate the symptoms of GAD. The PSWQ will be used to measure the tendency to worry. Statements are rated on a scale ranging from 1 to 5 and a higher total score indicates a greater tendency to worry. The psychometric qualities of the PSWQ with older adults are satisfactory [46]. The GAD-7 [23] will be used to assess the severity of GAD symptoms. GAD-7 statements are answered on a scale ranging from 0 to 3 and a higher total score suggests greater severity. Wild et al. [47] have shown the validity of the GAD-7 for older adults.

#### Secondary outcome measures

Measures will be used to evaluate changes in the psychological mechanisms targeted by treatment. Items on the Intolerance to Uncertainty Inventory (IUI [48]), the Negative Problem Orientation Questionnaire (NPOQ [49]), the Cognitive Avoidance Questionnaire (CAQ [50]), and the Why Worry Questionnaire (WWQ [51]) are all rated on scales ranging from 1 to 5. Higher total scores suggest higher intolerance to uncertainty (IUI), more negative attitudes toward problems (NPOQ), more likely use of cognitive avoidance (CAQ), and greater belief that worrying is useful (WWQ), respectively. All these instruments have been validated [48–51].

The Geriatric Anxiety Inventory (GAI [52]) evaluates anxiety symptoms in general. Depressive symptoms will be evaluated with the Geriatric Depression Scale (GDS [53]). The GAI and GDS have been designed specifically for an older population and their validity and reliability is well established. A higher total score for the GAI and GDS is indicative of greater severity of symptoms. The Insomnia Severity Index (ISI [54]) assesses sleep difficulties. Each statement is rated on a scale ranging from 1 to 4. A higher total score suggests more severe insomnia. Bastien et al. [55] demonstrated the validity and sensitivity of the ISI in older adults. The degree of functional deficit in different areas of activity (e.g. family life / domestic tasks) will be assessed with the Sheehan Disability Scale (SDS [56]). The validity of the SDS has been established [57]. Secondary outcomes will also include diagnosis of GAD, as assessed by the ADIS-5, and cognitive difficulties, as assessed by the T-MMSE.

#### Other outcome measures

A questionnaire will be used to collect feedback from participants and LPs about the treatment. They will respond to 19 statements covering various aspects of treatment (modules, readings, exercises, telephone sessions, workload, organization and duration of treatment, and its usefulness). All statements will be formulated positively (e.g., “the different treatment modules are relevant” and “readings are clear.”). Participants will rate their treatment experience while LPs will indicate their perception of treatment for each participant. Each statement will be evaluated on a four-point scale ranging from “strongly agree” to “absolutely disagree”. A fifth possible answer, “does not apply”, will also be provided. Participants and LPs will be invited to comment on each answer.

Recently researchers have shown that certain specific behaviors are associated with symptoms of generalized anxiety, including seeking reassurance, avoidance, control, procrastination, over-planning and checking [58, 59]. These behaviors, identified as factors that maintain anxiety, are likely to be targeted by our self-treatment through exercises designed to encourage the participant’s exposure to uncertainty and worrying situations. The Questionnaire of Behavioral Manifestations of Anxiety (QBMA; Brisson, M., Gossselin, P.: Questionnaire des manifestations comportementales liées à l'anxiété, unpublished) includes 24 items evaluating six behaviors related to generalized anxiety, namely reassurance seeking, avoidance, control, procrastination, overplanning and checking. Items are rated on a five point Likert scale. The validity and reliability will be investigated in conjunction with the conduct of this study.

Nine items were added during the study to assess anxiety associated with COVID-19 and its impacts on worry and anxiety. Five questions are framed as “yes or no” with an open-ended description of their answer choice whereas four items are rated on a five-point Likert scale.

#### Other measures

The Anxiety Literacy Questionnaire (A-Lit [60]) assesses mental health literacy specific to anxiety. Items are answered with one of three options – true, false or don’t know. Higher scores indicate higher mental health literacy of anxiety. The short version of Sarason’s Social Support Questionnaire (SSQ6 [61]) measures satisfaction and availability of appraisals of social support. The Treatment Motivation Questionnaire (TMQ [62]) measures the dimensions of an individual’s motivation for entering treatment. Specifically, it contains subscales measuring external and internal motivation as well as confidence in treatment outcome and help-seeking behaviors. The version used in the present study does not include the *Help seeking* subscale. Items are listed on a 7-point Likert scale ranging from 1 (not at all true) to 7 (very true). The Credibility/Expectancy Questionnaire (CEQ [63]) evaluates how each participant views the credibility of the treatment and the possible improvements that can be achieved from the treatment. The items are listed on a 9-point Likert scale, with a higher score indicating more trust in the treatment. Finally, the Working Alliance Inventory (WAI [64]) assesses the quality of the relationship between the participant and his LP. The WAI has two versions: one for the participant and another for the LP. Items are rated on a 7-point Likert scale, with higher scores indicative of greater alliance.

### Treatment

Treatment will be the same as used in our previous study [33]. All treatment material will be sent to participants by the site’s professional research assistant. Prior to beginning treatment, the LP will contact the participant, ensure that he has received the material, briefly explain how the treatment works, tell the participant that he can start and make an appointment with him for the first telephone session. Complete treatment duration will be 15 weeks and be based on a participant’s manual consisting of eight modules. The main components of treatment are relaxation, differentiation of the main types of worries, learning to tolerate uncertainty, questioning false beliefs about worries, problem solving, cognitive exposure, and planning pleasant activities. The content of the modules is presented in Table 1. Each module will be the topic of one to three telephone sessions with the LP, at the rate of one session per week. The participant will be asked to read the corresponding module each week and do the recommended exercises. The sessions with the LP will be limited in duration (30 min maximum) and the role of the LP will be restricted to providing support within treatment procedures, namely motivational support (motivate the participant to continue treatment), emotional support (make the participant feel he is understood), and technical support (track activities done and to be done and answer technical questions related to treatment). Each session will follow an identical sequence: it will begin with a summary of the activities completed since the last session, followed by answering participant’s questions and finishing with planning the next week’s activities. LPs will not be allowed to practice psychotherapy in any form including applying techniques to modify participant’s beliefs and behaviors such as described in the treatment manual.

**Table 1:** Content of the Modules

Module Title	Number of sessions	Content
1. Therapy goals and basic components of anxiety	1	• Therapy characteristics • Key elements for success • The two types of worries
2. Relaxation	1	• Abdominal breathing • Progressive muscle relaxation • Imagery
3. Tolerating uncertainty	3	• Exposure to uncertainty
4. Enjoying yourself	2	• The relation between anxiety and depression • Identification of pleasant activities • Planning pleasant activities
5. Correcting false beliefs	1	• Questioning false beliefs about worrying
6. Effective problem solving	3	• Steps for effective problem solving
7. In my mind … cognitive exposure	3	• Exposure to problems with uncertain outcomes
8. Relapse prevention	1	• Summary of learnt techniques • Review of gains • Relapse prevention advice

### Training and supervision of LPs

 LPs will be bachelor’s degree students in psychology or a related field and will have no post-graduate training in a specialized mental health program. They will be recruited through interviews to assess communication and interpersonal skills. Training will be divided into 2 successive steps spread over a period of one to 2 weeks. The first step (6 hr) will consist of readings on GAD in older adults, basic helping relationships skills, and the GSH-CBT participants’ manual. The second step (4.5 hr) will consist of didactic training, including demonstrations, role plays, and feedback,  on the support to be provided by LPs to participants. LPs will be informed of the procedures used for ongoing biweekly supervision of their work by a licensed psychologist, namely authors PL, PG, and SG, depending on study site. They will also be informed of the crisis protocol to be applied in the event of a worsening of the participant’s condition (e.g., suicidal risk, severe depressive symptoms). This protocol will involve reporting the situation immediately to the psychologist, who will follow up with the participant. In addition, a meeting with all LPs involved in the study will be held once a year to discuss practical issues and hence maximize cohesion between the three sites. Similar meetings will be held with research assistants conducting assessments to ensure that they follow the evaluation process and to answer their questions.

### Procedure

After initial telephone screening and clinical psychological assessment, eligible individuals will sign the consent form and answer self-administered questionnaires. The schedule of enrolment, interventions, and assessments is shown in Table 2. Participants will complete self-administered questionnaires at home and will have the choice between paper or online questionnaires. Persons who are found not eligible for the study but who would like help with a personal difficulty will be referred to an alternative resource.

**Table 2:**
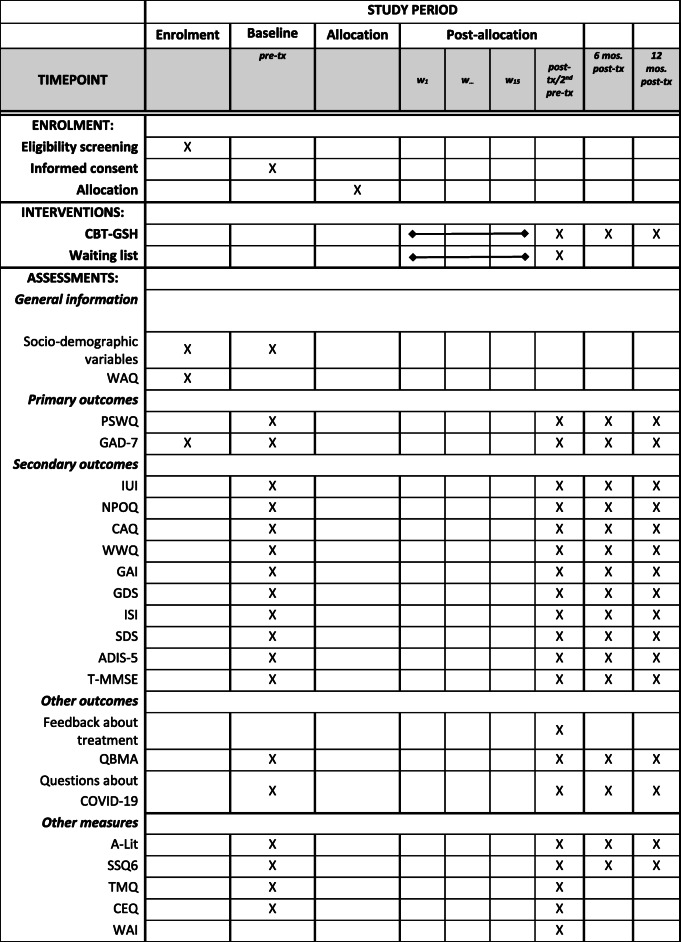
Schedule of Enrolment, Interventions, and Assessments

*ADIS-5* Anxiety and Related Disorders Interview Schedule for DSM-5, *A-Lit* Anxiety Literacy Questionnaire, *CAQ* Cognitive Avoidance Questionnaire, *CEQ* Credibility/Expectancy Questionnaire, *GAI* Geriatric Anxiety Inventory, *GDS* Geriatric Depression Scale, *ISI* Insomnia Severity Index, *IUI* Intolerance to Uncertainty Inventory, *NPOQ* Negative Problem Orientation Questionnaire, *PSWQ* Penn State Worry Questionnaire, *QBMA* Questionnaire of Behavioral Manifestations of Anxiety, *SDS* Sheehan Disability Scale, *SSQ6* Short version of Sarason’s Social Support Questionnaire, *T-MMSE* Telephone version of Mini-Mental State Examination, *TMQ* Treatment Motivation Questionnaire, *W* week during treatment or waiting period, *WAI* Working Alliance Inventory, *WAQ* Worry and Anxiety Questionnaire, *WWQ* Why Worry Questionnaire

A block randomization procedure stratified on diagnosis will be used. The randomization sequence will be determined prior to recruitment by the project coordinator. Block size will be fixed at 4, all possible balanced combinations of assignments within a block will be identified, and blocks will be randomly chosen to determine participants’ assignment to groups. Randomly assigned conditions will be sealed in envelopes labelled with participant codes and opened by the project coordinator following completion of the pre-treatment assessment. Group assignment will be done separately for participants with threshold and subthreshold GAD. Participants assigned to the experimental group will begin treatment immediately after the pre-treatment assessment. Participants assigned to the control group will have a 15-week wait period, during which they will receive a 15-min telephone call from the local professional research assistant on four occasions separated by four-week intervals. The purpose of these calls is to encourage the participant to persevere until the start of treatment and to identify potential negative effects that could be associated with the waiting period. For the experimental group participants, assessments will take place at pre- and post-treatment and at 6 and 12 months post-treatment. For control group participants, three assessments are planned: two at pre-treatment (before and after the waiting period) and one after receiving treatment (post-treatment). All participants will receive financial compensation ($20 CAD) for each of the post-treatment and follow-up assessments to support participation in post-treatment assessments.

### Adherence to treatment

At the end of each telephone session, LPs will take note of the pages read and the exercises completed by the participant during the previous week. They will also take note of the content covered during the session. Furthermore, LPs will evaluate each of the following aspects on a scale ranging from 0 (very weak) to 8 (very good): (1) degree of participant implication with regards to self-treatment, and (2) overall assessment of the quality of the collaboration perceived during the interview.

The degree to which LPs have adhered to the specified tasks assigned to them will also be assessed. All sessions for each participant will be recorded and divided in three parts. An equivalent number of sessions in each part will be randomly selected to obtain 25% of sessions. The integrity of these sessions will be verified by an independent evaluator using a first list of acceptable procedures (e.g., proving motivational support) and a second list of procedures that should not be used (e.g., recommending thought stopping and distraction). The first list will only include procedures presented during training. The evaluator will record the time spent on each procedure. The time spent on procedures in the first list compared to the total time of the session will be calculated.

### Data management and analysis

The principal investigators (PL, PG, and SG) will have access to all trial data. To protect the identity of participants and confidentiality of information, participants will be identified by a code number. Data obtained by paper questionnaires will be kept in a secure file. Data in electronic format will be kept on a secure server accessible to the research team only. Research assistants will double-entry data in paper format in order to detect and correct data entry errors. Data obtained from online questionnaires will be added to the data files.

SPSS for Windows will be used for statistical analyses. Missing data will be handled using multiple imputation. Descriptive analyses of study variables will be carried out. Analyses will follow intention-to-treat principles (i.e., data from all participants included in the study will be analyzed according to the randomization scheme). The pre- and post-treatment data will be used to compare the experimental and control groups. Repeated measures analysis of variance (ANOVA) will be performed on most outcome measures. Simple effects for group and time and group by time interactions will be analysed. Between group comparisons will include remission rate based on GAD diagnosis using the chi square test and clinically significant change which will be examined according to procedures described by Jacobson et al. [65, 66]. Change on the Sheehan Disability Scale will be analysed according to Sheehan and Sheehan’s [67] criteria. In order to determine whether the effects of the treatment are sustained over time, the post-treatment, 6 and 12-month data of the experimental group will be used. Repeated measures ANOVA will be carried out on the different variables and followed by contrast analyses. All model assumptions will be checked. Participants’ and LPs’ perception of treatment will be analyzed by calculating the frequency of each answer choice for the various statements and by summarizing comments.

The relationship between variables and improvement during treatment will be examined by combining the two groups’ pre- and post-treatment data. A change ratio will be calculated for each measure of GAD symptoms in order to obtain a score representing treatment response. Univariate analyses will be performed first using Pearson correlations for continuous variables and ANOVAs for categorical variables. Subsequently, variables that are significantly related to a change on each GAD symptom measure will be retained for a stepwise multiple regression analysis on this measure to identify the best predictors of change.

### Dissemination

We plan to disseminate the results of the study to public health policy makers, the general public, the scientific community and mental health professionals by using various strategies including press releases, presentations at conferences, and articles in scientific and professional publications.

## Discussion

This study aims to provide evidence of the efficacy of GSH-CBT guided by LPs for treating GAD in older adults. This approach aims to facilitate access to psychological treatment for GAD by reducing patient travel and by involving trained and supervised LPs, rather than mental health professionals, for guiding patients.

This study presents certain issues that deserve to be highlighted. Firstly, all stages of the protocol, including assessments and sessions with LPs, are done remotely and communication with participants is essentially done by telephone. We are aware that telephone exchanges with participants can result in the loss of clinically useful information. For example, the use of the telephone does not allow to see the participant and thus access non-verbal cues. Nevertheless, we opted for a completely remote experimentation in order to be consistent with the problem at the source of the project, namely the difficulty of access to psychological treatments by older persons. We also made this decision on the basis of our experience [33] and that of other researchers who have also used the telephone successfully as the main means of communication with older adults with GAD [68]. We considered but ruled out the idea of using a televideo system to address the limitations of the telephone. There is still a generational digital divide [69] and we wanted to avoid having to exclude participants who did not have access to the necessary equipment or had low levels of computer literacy.

A second issue is the involvement of LPs to guide participants through treatment. Again, precedents suggest the feasibility of this approach [31]. However, legal aspects had to be taken into consideration when defining the tasks of LPs. Psychotherapy is a reserved act in several jurisdictions, including in Canada’s province of Quebec where the study takes place and where the practice of psychotherapy is reserved for psychologists, physicians and persons holding a psychotherapist’s permit. However, there are interventions, such as providing support, which, although similar, do not constitute psychotherapy and are therefore not acts reserved for these professionals. LPs are therefore not trained to do psychotherapy (in fact, an important part of the training consists of specifying what they can and cannot do), but rather to develop skills to accompany older patients receiving CBT-GSH.

A third issue is the implications of the COVID-19 pandemic for the continuation of the study. Like many researchers, we were confronted with this major situation and had to think about the impact on the anxiety of our participants. We also wondered about their ability to continue treatment, or at least some of its components. For example, the module on tolerance to uncertainty includes an exercise in which the patient is asked to expose himself to a situation with an uncertain outcome and some examples refer to an outing (for example, traveling to visit a distant sister despite the risk of a car accident). The confinement of the population in the spring of 2020 has considerably reduced the possibility of leaving home and therefore could make it more difficult to carry out this exercise. Fortunately, we did not observe a marked increase in our participants’ worrying and anxiety related to the pandemic and confinement and they were generally able to continue treatment as planned. Nevertheless, we decided to include a few questions about COVID-19 in our measures. In addition, this experience has shown us the usefulness of the intervention in facilitating access to mental health care by older adults in the context of pandemic and containment.

GSH-CBT is consistent with a stepped care model and has the potential to be a more accessible intervention than psychotherapy. CBT for GAD has been found to be less expensive when delivered by LPs than by licensed providers [27] which suggests an additional benefit of LP-guided treatment. If GSH-CBT is effective, we can consider that it could be implemented more broadly and through an expanded workforce to provide much-needed mental health treatment to many older adults.

## Data Availability

Not applicable.

## Abbreviations

ANOVA: Analysis of variance
ADIS-5: Anxiety and Related Disorders Interview Schedule for DSM-5
A-Lit: Anxiety Literacy Questionnaire
CAQ: Cognitive Avoidance Questionnaire
CEQ: Credibility/Expectancy Questionnaire
DSM-5: Diagnostic and Statistical Manual of Mental Disorder fifth edition
GAD: Generalized Anxiety Disorder
GAI: Geriatric Anxiety Inventory
GDS: Geriatric Depression Scale
GSH: Guided self-help
GSH-CBT: Guided self-help based on the principles of cognitive-behavior therapy
ISI: Insomnia Severity Index
IUI: Intolerance to Uncertainty Inventory
LPs: Lay providers
T-MMSE: Mini-Mental State Examination, telephone version
NPOQ: Negative Problem Orientation Questionnaire
PSWQ: Penn State Worry Questionnaire
QBMA: Questionnaire of Behavioral Manifestations of Anxiety
SSRIs: Selective serotonin reuptake inhibitors
SDS: Sheehan Disability Scale
SSQ6: Social Support Questionnaire, short version
TMQ: Treatment Motivation Questionnaire
WWQ: Why Worry Questionnaire
WAI: Working Alliance Inventory
WAQ: Worry and Anxiety Questionnaire
